# Knowledge, attitude and willingness of different ethnicities to participate in cadaver donation programs

**DOI:** 10.1371/journal.pone.0229529

**Published:** 2020-03-12

**Authors:** Xiang Zhang, Li Peng, Lan jiang Li, Wei Fan, Jie Deng, Xiaohan Wei, Xing Liu, Zhongming Li

**Affiliations:** 1 Experimental Demonstration Center, College of Basic Medical Science, Kunming Medical University, Kunming, Yunnan, China; 2 College of Forensic Medicine, Kunming Medical University, Kunming, Yunnan, China; 3 Department of Human Anatomy and Tissue Embryology, College of Basic Medical Science, Kunming Medical University, Kunming, Yunnan, China; University of Toronto, Rotman School, CANADA

## Abstract

Knowledge, attitude and willingness of ethnic minorities in China towards cadaver donation programs were assessed. Questionnaire and interviews were conducted to investigate Yi, Bai, Hani, Dai and Han ethnicities. Educational level and per capita income of ethnic minorities were lesser than those of Han ethnicity (*p*<0.01). Agriculture was the primary occupation and proportions of technical personnel and public officials was lesser among ethnic minorities (*p*<0.01). Surveyed ethnic minorities universally practice religious traditions, Bai and Dai ethnicities practice Buddhist beliefs also (*p*<0.01). Knowledge of Yi, Bai, Hani and Dai ethnic respondents was lesser than those of Han ethnicity (*p*<0.01). Over 83.8% of Yi, Bai, Hani and Dai ethnicity residents were unwilling to register for body donation programs with receiving a driver's license (*p*<0.01). Less than 46.9% of ethnic minorities supported use of honorary certificates (*p*<0.01). Ethnic minorities were supportive of financial compensation for body donations and denied that financial compensation led to the commercialization of cadaver donation (*p*<0.01, p<0.01). Willingness of ethnic minorities to participate in cadaver donation programs was primarily related to religious beliefs (*p*<0.01), economic status (*p*<0.01). Knowledge, attitude and willingness of ethnic minorities to participate in cadaver donation programs were markedly different from those of Han ethnicity, and the religious belief and economic status played a decisive role. To increase participation, programs based on respecting religious belief should be developed to support improvements in economy, education, medical care and social security system.

## Introduction

Yunnan, China is a multi-ethnic and religious province with a population of 17.46 million ethnic minorities, accounting for 38% of the total population in Yunnan province. The four most abundant ethnic minorities in Yunnan province are Yi, Bai, Hani and Dai ethnicities, each with more than 1 million citizens and accounting for over 50% of all ethnic minorities in Yunnan province When compared amongst each other and with the prominent Han ethnic group, there are differences in life style, genetics, social culture and religious practices [1–4].

In 2018, there were 151 registered body donations for teaching and research in medical and anatomical sciences in Yunnan province, less than 1 per 1000000 residents and is drastically lesser than other countries where rates can range from 30%-80%. And in some instances, the number of people willing to donate body is much more than the actual needs of medical schools[5–6]. Rates of body donation in other provinces of China are greater, such as in Shenzhen, Guangdong Province where registrations exceeded 1000 for scientific purposes in 2015 and far exceeded total registrations in Yunnan Province over the past 30 years [5, 7]. Furthermore, over 99% of body donations in Yunnan Province are Han ethnicity [5], highlighting the discrepancy between registered body donations of different ethnicities and religions as ethnic minorities account for 50.2% of the population. Therefore, a greater understanding of the drivers and views surrounding body donation among ethnic minorities might help improve rates of donation in ethnic minorities region.

## Objects and method design

### Interviews

Using a stratified unequal probability second-order clustering approach, eight autonomous prefectures of Yunnan Province were selected for analysis. Sites included Gejiu City of Honghe Hani ethnicity and Yi ethnicity Autonomous Prefecture (Hani ethnicity and Yi ethnicity), Jinhong City of Dai ethnicity Autonomous Prefecture of Xishuangbanna (Dai ethnicity), Dali Bai ethnicity Autonomous Prefecture (Bai ethnicity) and Jinning District of Kunming City (Han ethnicity). In Baohe township of Honghe Hani and Yi autonomous prefecture, Yi people in the region account for 73.27% of the population. Adjacent to Baohe township, Hani people in the Baohua township makes up 84% of the population. Gasa town of Xishuangbanna Dai autonomous prefecture is the main residential area of Dai people, contributing to 85.7% of the population. Diannan Town of Dali Bai autonomous prefecture is a densely populated area of Bai people, making up 88.12% of the population. However, Jinning District (Plain) in Kunming City is a region where the Han people is concentrated, with 88.4% Han residents. The aforementioned research areas are all inhabited by ethnic minorities. The survey of regional ethnic minorities can reflect the knowledge, attitudes, and willingness of different ethnicities regarding the donation program.

### Survey tools and survey respondent

The questionnaire included instruction, the basic data of the interviewees and the main content of the questionnaire. Instruction included the purpose, significance and method. The basic data included gender, age, educational level, monthly income and religious belief. The subjects of the questionnaire included cadaver donation knowledge, cadaver donation attitude and cadaver donation willingness.

The questionnaire consisted of 18 variables. Using the Kendall sample size estimation method, a sample size 20 times the number of variables was selected (i.e. 360). To minimize variability, sample size was expanded by 15%, with an additional 6 per group to limit the impact of questionnaires not being returned (i.e. 420 per group). Overall, 1680 questionnaires were distributed. Inclusion criteria for residents included being aged 18 to 75, understanding purpose of the study and volunteering to participate. In this study, we studied ethnic minorities aged over 18 years (Han Chinese also follows the protocol). Investigators conducted the survey after well-trained by the local Disease Control Center staff. This survey obtained the support of the subdistrict office staff and social service volunteers. Social service volunteers have built trust because of their long-term residence in the same community. Furthermore, with the assistance of subdistrict officers and the volunteers, verbal consent had been obtained by investigators before the survey. Afterwards, the investigators assisted the subjects in reading the questionnaire and explained to subjects individually if they have questions, and then stored the questionnaire appropriately once finished, in order to respect the autonomy and protect the confidentiality of subjects. In addition, if the subject was not at home when the researchers visited, a follow-up visit will be scheduled, and the subject’s family will be informed. Through such approaches, the survey achieved a 100% response rate.

### Statistical analysis

Data were analyzed using the Statistical Package for Social Sciences (SPSS23). Age was summarized as mean ± standard deviation, and results of questionnaire were described using quantitative (n) and constituent ratios (%). The Chi-squared test (χ^2^) was used to assess difference among groups where p<0.05 was used to identify statistically significant differences.

### Ethical considerations

The survey’s questionnaire was reviewed and approved by the Ethics Committee of Kunming Medical University(KMEC). KMEC agreed to utilize verbal consent process instead of obtaining the subject’s signature due to the concern of privacy issues.

## Results

### Demographic data

No difference was observed for age or gender among different ethnicities who responded to the questionnaire (*p*>0.05). When compared with respondents of Han ethnicity, the level of educational of ethnic minorities was lesser, proportion of illiterate people was larger, and total number of respondents who attended higher education was lesser (χ^2^ = 36.34, *p*<0.01). The proportion of agricultural workers was larger among minority ethnic groups when compared to those of Han ethnicity, and proportions of respondents occupying technical roles and public officials was lesser (χ^2^ = 587.64, *p*<0.01) (Table 1). More than 50.0% of the income of ethnic minorities was at a lower level (monthly income<2000 CNY) and the proportion is much higher than that of Han ethnicity low-income respondents (χ^2^ = 279.02, *p*<0.01). All ethnic minorities actively practice different religious beliefs and some had mixed beliefs. Respondents identifying as Han followed the culture of Confucia and practices of Buddhism partially (χ^2^ = 2783.95, *p*<0.01).

**Table 1:** General demographic data of respondents (n = 1680).

	Han ethnicity	Yi ethnicity	Bai ethnicity	Hani ethnicity	Dai ethnicity	χ^2^	*p*
	n	n	n	n	n		
Gender							
Male	214	211	216	197	217	2.53	0.639
Female	206	209	204	223	203		
Age							
18–25	138	152	128	134	142	4.27	0.831
26–60	172	160	178	174	176		
>61	110	108	114	112	102		
Educational level							
Uneducated	28	64	54	70	48	36.34	0.000
Primary school-senior high school	362	346	353	337	356		
At and above junior college	30	10	13	13	16		
Occupational differentiation							
Agricultural personnel	211	403	396	402	400	587.64	0.000
Technician	172	12	14	13	14		
Public officers	37	5	10	5	6		
Monthly income (CNY)							
<2000	61	247	263	238	221	279.02	0.000
2000–5000	287	152	138	165	173		
>5000	72	21	19	17	26		
Traditional belief							
Confucian culture	358	0	0	0	0	2783.95	0.000
Buddhism	62	0	32	0	294		
Other religion	0	420	388	420	126		

### Knowledge of donation programs

Overall, 86.2% of ethnic Yi respondents, 87.1% of ethnic Bai respondents, 84.3% of ethnic Hani respondents and 86.7% of ethnic Dai respondents reported no knowledge of the body donation, which was significantly higher than respondents of Han ethnicity (32.6%) (χ^2^ = 520.5, *p*<0.01). In addition, next to 12 respondents of Han ethnicity and 1 respondent of Bai ethnicity, there were essentially no precedent of cadaver donation register around ethnic minority groups (χ^2^ = 43.04, *p*<0.01) (Table 2). Furthermore, 94.3% of ethnic Yi respondents, 93.3% of ethnic Bai respondents, 94.8% of ethnic Hani respondents and 93.8% of ethnic Dai respondents reported a lack of understanding of the procedures and management department, which was higher than among ethnic Han respondents (59.8%) (χ^2^ = 354.1, *p*<0.01).

**Table 2:** Knowledge of body donation program among different ethnicities.

	Han ethnicity	Yi ethnicity	Bai ethnicity	Hani ethnicity	Dai ethnicity	χ^2^	*p*
	n (%)	n (%)	n (%)	n (%)	n (%)		
Awareness of body donation							
Unknown	137 (32.6)	362 (86.2)	366 (87.1)	354 (84.3)	364 (86.7)	520.5	0.000
Partial known	173 (41.2)	34 (8.1)	32 (7.6)	46 (10.9)	30 (7.1)		
Comparative known	110 (26.2)	24 (5.7)	22 (5.3)	20 (4.8)	26 (6.2)		
Is there someone around who have donated their bodies?							
Yes	12 (2.9)	0 (0)	1 (0.2)	0 (0)	0 (0)	43.04	0.000
No	408(97.1)	420 (100)	419 (99.8))	420 (100)	420 (100)		
Procedure and management of body donation							
Known	169 (40.2)	24 (5.7)	28 (6.7)	22 (5.2)	26 (6.2)	354.1	0.000
Unknown	251 (59.8)	396 (94.3)	392 (93.3)	398 (94.8)	394 (93.8)		

### Attitudes towards body donation program

Overall, 92.0% of ethnic Yi respondents, 93.2% of ethnic Bai respondents, 91.1% of ethnic Hani respondents, 92.8% of ethnic Han respondents and 95.4% of ethnic Dai respondents consider body donation as beneficial to the progress of medicine research and anatomical sciences (χ^2^ = 7.828, p>0.05) (Table 3). However, only 2.9% of ethnic Yi, 2.1% of ethnic Bai, 3.4% of ethnic Hani and 6.2% of ethnic Dai respondents were willing to register for the body donation program at the time of obtaining a driver’s license, and were significantly lesser when compared to respondents of Han ethnicity (18.9%,χ^2^ = 315.83, *p*<0.01). Over 95.2% of ethnic Han respondents believed that body donation should be recognized by issuance of honorary certificates from the government, whereas only 32.6% of ethnic Yi, 29.9% of ethnic Bai, 31.4% of ethnic Hani and 46.9% of ethnic Dai respondents preferred receiving a government issued certificate (χ^2^ = 539.97,*p*<0.01). Fifty-five percent of ethnic Han respondents believed that financial compensation for body donation was wrong whereas only 7.9% of ethnic Yi, 8.1% of ethnic Bai, 9.9% of ethnic Hani and 12.1% of ethnic Dai respondents felt similarly (χ^2^ = 459.20,*p*<0.01). Additionally, 87.6% of ethnic Han respondents worried that financial compensation would increase commercialization of body donations, and was significantly different from those of ethnic Yi, Bai, Hani and Dai respondents, respectively (12.4%,10.4%,11.9% and 16.9%; χ^2^ = 913.47,*p*<0.01). Furthermore, the responses of ethnic Dai respondents were different for those of Yi, Bai and Hani ethnicities for the past 4 questions with no significant difference and were similar to ethnic Han respondents.

**Table 3:** Comparison of attitudes towards body donation among different ethnicities.

	Han ethnicity	Yi ethnicity	Bai ethnicity	Hani ethnicity	Dai ethnicity	χ^2^	*p*
	n (%)	n (%)	n (%)	n (%)	n (%)		
Body donation = noble cause							
Yes	390 (92.8)	386 (92.0)	390 (93.2)	382 (91.0)	400 (95.4)	7.828	0.450
No	13 (2.7)	12 (2.9)	10 (2.2)	16 (3.9)	6 (1.4)		
Uncertain	17 (4.5)	22 (5.1)	20 (4.6)	22 (5.1)	14 (3.2)		
Driver’s license + signing of petition for body donation							
Yes	79 (18.9)	12 (2.9)	9 (2.1)	14 (3.4)	26 (6.2)	315.83	0.000
No	240 (57.2)	386 (91.9)	401 (95.5)	390 (92.8)	352 (83.8)		
Uncertain	101 (23.9)	22 (5.2)	10 (2.4)	16 (3.8)	42 (10)		
Body donation should be issued by the Government with a certificate of honour.							
Yes	400 (95.2)	137 (32.6)	126 (29.9)	132 (31.4)	197 (46.9)	539.97	0.000
No	4 (1)	249 (59.3)	263 (62.8)	259 (61.7)	178 (42.3)		
Uncertain	16 (3.8)	34 (8.1)	31 (7.3)	29 (6.9)	45 (10.8)		
Financial compensation tarnishes body donation							
Yes	231 (55)	33 (7.9)	34 (8.1)	42 (9.9)	57 (12.1)	459.20	0.000
No	149 (35.5)	334 (79.5)	341 (81.3)	331 (78.9)	311 (74.1)		
Uncertain	40 (9.5)	53 (12.6)	45 (10.6)	47 (11.2)	52 (13.8)		
Financial compensation causes commercialization of body donation							
Yes	368 (87.6)	52 (12.4)	57 (10.4)	50 (11.9)	71 (16.9)	913.47	0.000
No	33 (7.8)	293 (69.8)	287 (68.3)	308 (73.3)	265 (63.1)		
Uncertain	19 (4.6)	75 (17.8)	76 (21.3)	62 (14.8)	84 (20)		

### Comparison of residents’ willingness to register with the cadaver program

Because of the difference of religious belief, there are significant differences in the willingness of body donation among different ethnicities (χ^2^ = 37.46, p< 0.01). The donation willingness rate of Han ethnicity affected by Confucian culture is 10.9%, but the donation willingness rate of Han ethnicity people who believe in Buddhism increases to 27.5%. Yi ethnicity, Bai ethnicity, Hani ethnicity are trapped in religious belief, the donation willingness rate is as low as 2.1,2.0 and 1.9%, respectively. Within Bai ethnicity, the donation willingness rate of Buddhist residents affected by religious beliefs is lower than that of Han ethnicity who believe in Buddhists, with the value of only 3.1%. Dai ethnicity belief shows the characteristics of mixed preference between other religion and Buddhist belief, the donation willingness rates of those with mainly other religious belief and those with mainly Buddhist belief are higher than those of other ethnic minorities, with the value of 2.3% and 6.8%, respectively.

Among ethnic Han respondents who were categorized into the education groups; uneducated, primary to high school, or at and above junior college, the willingness to donate was 7.1, 12.7 and 23.3%, respectively (Table 4). These results suggest a positive correlation between level of education and willingness to donate. Among ethnic minorities, willingness to donate of uneducated people was highest at 9.3, 11.1, 5.7 and 14.5% for respondents of Yi, Bai, Hani and Dai ethnicities, respectively. Overall ethnic minority willingness to donate was negatively correlated with level of education (χ^2^ = 40.34,*p*<0.01). The willingness to donate of ethnic Han respondents who work in agriculture (5.6%) was least among occupations and was lesser than technical personnel (19.7%), and public officials (27.0%). Willingness to donate for respondents occupying positions as technical personnel and public officials was 0% for all ethnic minorities(except Dai ethnicity) and was 2.2,2.3,1.9 and 5.5% for respondents occupying positions as agricultural personnel of Yi, Bai, Hani and Dai ethnicities, respectively (χ^2^ = 32.65,*p*<0.01). Among ethnic Han respondents, willingness to donate for low-income people (1.6%) was lesser than that of high-income people (20.8%). Respondents of Yi, Bai and Hani ethnicities had lesser willingness to donate in the high-income group (0%) when compared to the lower income group, respectively (3.2, 3.0 and 2.9%) (χ^2^ = 26.54,*p*<0.01). The willingness to donate of ethnic Dai respondents was higher than that of Yi, Bai and Hani ethnicities when considering level of education, occupation or monthly income.

**Table 4:** Comparison of the willingness to body donation among different ethnicities.

	Han ethnicity	Yi ethnicity	Bai ethnicity	Hani ethnicity	Dai ethnicity	χ^2^	*p*
	%	%	%	%	%		
Traditional belief (%)							
Confucian culture	10.9	0	0	0	0	37.46	0.000
Buddhism	27.5	0	3.1	0	6.8		
Other religion	0	2.1	2.0	1.9	2.3		
Educational level (%)							
Uneducated	7.1	9.3	11.1	5.7	14.5	40.34	0.000
Primary school-senior high school	12.7	0.8	0.9	1.2	4.2		
At and above junior college	23.3	0	0	0	6.3		
Occupational differentiation (%)							
Agricultural personnel	5.6	2.2	2.3	1.9	5.5	32.65	0.000
Technician	19.7	0	0	0	7.1		
Public officers	27.0	0	0	0	0		
Monthly income (CNY) (%)							
<2000	1.6	3.2	3.0	2.9	6.3	26.54	0.001
2000–5000	13.9	0.6	0.7	0.6	4.6		
>5000	20.8	0	0	0	3.8		

## Discussion

From 1998 to 2018, the number of medical students in ethnic regions increased 12-fold from 300 to 3600, while the number of body donors increased from 5 to 10, only doubling in the past two decades and lagging far behind the growth rate of medical students. The ratio of medical students to body donors raised from 60 to 1 in 1998 to 360 to 1 in 2018. The huge gap between the number of donated cadaver and the actual demand severely restricted the anatomical science education and scientific research, hindered the cultivation of medical students, and impeded the development of health care services in ethnic minority areas. [7].

The awareness of body donation of ethnic minorities is less than 15.7% (partial known+comparative known), which is much lower than the cognitive level of Han ethnicity (67.2%). There has never been body donation behavior around ethnic minorities (except 0.2% Bai ethnicity), which is far below the Han ethnicity of 2.9%. The low level of donation has also led to a lack of access to body donation for ethnic minorities. Only less than 6.7% of ethnic minority interviewees know about body donation procedures and management departments, which is not only far below the awareness rate of Han ethnicity (40.2%), but also far from the survey results of 21.93%-75% about knowledge of body donation program in China and abroad [8]. The low-level awareness rate is related to the lack of popularization of body donation information, and the superficial status of cognitive breadth and depth is not only related to the low educational level in ethnic minority areas, limited sources of body donation knowledge, but also related to the lack of publicity work in government agencies. In summary, ethnic minority residents not only have no way to know the knowledge of body donation, but also have the possibility of giving up donation due to “no way to donate” [9–11].

Studies had reported that 84.7% of medical professionals and students in China agreed that the body donation is a noble act. However we found 91% of minority considered body donation is respectful, it indicates that the quality of citizens of regional ethnic minorities in their attitude towards body donation is relatively high. It is suggested that the relevant institutions should take body donation knowledge as universal education for the quality of citizens, so as to change the ethical concept of death of ethnic minorities and lay the foundation for the follow-up work of body donation [12–15].

In 2016, 1937 ethnic drivers were killed in traffic accidents in the region [16], but there was no body donation associated with these deceased drivers. More than 83.8% of ethnic minorities hold opposition to the concept of combining the application for a driver’s license with the signing of cadaver donation procedure. The low level is due to that they misunderstand that the signing cannot go back on their word. The former is related to the lack of public awareness of body donation policy, suggesting that the government is quite absent in the related publicity work, while the latter is related to the traditional ethics of death, and the ideological infiltration is deep-rooted, so it is difficult to change overnight [17].

As high as 95.2% Han ethnicity people believe that the government should issue relevant certificates to body donor, but less than 46.9% of ethnic minorities agree this, indicating that Han ethnicity attaches more importance to social identity and spiritual encouragement of body donation. The data of 30-year body donation show that about 12% body donors had financial distress before they died [18]. The economy in ethnic areas is relatively backward, the level of social security for ethnic minorities is relatively low, and donors often have financial difficulties. The implementation of reasonable financial subsidies will help alleviate the plight of donors and their families. Therefore, more than 74.1% ethnic minorities do not agree that financial compensation will defile body donation. In the absence of basic survival security, requiring ethnic minorities to assume additional civic responsibility (body donation) is more likely to cause sensitivity and resentment of ethnic minorities. It is difficult for individuals or regions where the basic survival rights and interests could not be fully guaranteed to breed moral role models for feedback society [8, 19–21]. Therefore, more than 63.1% ethnic minorities deny that financial compensation will accelerate the commercialization of body donation.

Influenced by Confucian culture, Han ethnicities believe that a person’s soul is attached to their body. In addition, they believe that the soul is attached when living but leaves following death and travels to soul gathering areas (heaven or hell). Therefore, there is no subordination between the body and the soul. Surveyed respondents of Han ethnicity placed a greater emphasis on social responsibility, similarly rates of body donation willingness were greatest (10.9%). In addition, a large number of Buddhist believers exist among ethnic Han residents. As they have baptized according to Buddhist beliefs, people of Han ethnicity, who have been exposed to Confucian culture over time, are less concerned for physical integrity and their willingness to donate their bodies has increased (27.5%) [22–26].

Yi, Bai and Hani ethnicities are regional ethnic minorities with populations greater than one million, accounting for 48.2% of all ethnic minorities in the region. The religious beliefs of Yi, Bai and Hani ethnicities, including worship of nature, totems and ancestral, have strong religious characteristics. Among them, worship of ancestors is at the core of their beliefs, believing that the deceased continue to live in the underworld, with similar social relations and needs as their life on earth. Accordingly, living relatives fear that the deceased might interfere with the lives of future generations if they do not provide adequate funerals. In addition, it is hoped that the deceased will guide and protect future generations throughout life. Therefore, living people are fearful of the deceased. Due to their beliefs related to funeral etiquette (the way a body is handled) and how it is related to prosperity of future generations, the willingness to donate cadavers of Yi, Bai and Hani ethnicities is low (2.1% or less) [27–29].

The Dai ethnicity in Yunnan (1.14 million), account for 7.2% of minority populations and have beliefs of Southern Upper Buddhism. Influenced by Buddhism, Dai ethnicity believe that life transforms into different forms, whereby ending of the present life form is followed by the beginning of a new life form. Therefore, death is both an end and a beginning. As an important psychological construct, Buddhist beliefs meet the spiritual needs of Dai individuals allowing them to transcend fear of life and death, alleviating anxiety when faced with death, thus ensuring participants are more comfortable when contemplating what is done with a body following death [30–32]. Similarly, results of the questionnaire demonstrated higher rates of acceptance for the body donation program when compared to respondents of Yi, Bai and Hani ethnicities, although these results were not statistically significant. The Bai ethnicity residents mainly believe in more religious beliefs. However, influences of Buddhist beliefs, result in residents of Bai ethnicity having a dual nature. When compared with simple religious beliefs such as ancestral worship, concern of people who have mixed religious beliefs wore accepting of the cadaver donation program and had greater willingness to donate (6.8%).

Previous studies on donation of corpses in China have suggested that highly educated donors were more receptive to the idea of body donation [33,34]. In addition, people of higher education, Technician/Public officers, high-income population derive more benefits from social security due to more incomes, and generally have a higher sense of social responsibility [35–40].However respondents of Yi, Bai, Hani or Dai ethnicity demonstrated opposite tendencies and those characterized by lesser income and education were more active participants in the body donation program. The reasons are respondents who are not economically advantaged, or are lower on the social ladder (education, career), are under increasingly financial pressure cannot perform their basic needs and thus cannot practice their religious beliefs, in order to obtain uncertain economic assistance after cadaver donation. Therefore, there is a need to characterize the effect of financial compensation for body donation in marginalized populations.

When considering the influence of more religious systems, a lack of rational knowledge of death is a main contributing factor to restricted rates of participation in body donation programs among ethnic minorities. In addition, lesser economic achievement and levels of education among ethnic minorities played important roles. Ethnic minorities mainly live in remote mountain areas with geographical space barriers and harsh natural conditions, which makes the level of productivity of ethnic minorities lag and restricts regional economic development. The local education investment is constricted by the weak economy, and the minority residence is extremely scattered due to the geographical separation of the mountains. As a result, the increase in cost of individual education impedes the development of education on a large scale. To sum up, economic backwardness and low education level are not the inherent labels of ethnic minorities, but the common dilemma faced by all ethnical populations living in remote mountain areas.

Overall, we should avoid judging from the point of view of moral strangers with the prejudice of “traditional concepts and feudal superstitions”. Participation in body donation programs is influenced by a number of factors including religious beliefs, economic conditions, education and informed consent, therefore it is important to address discrepancies of income, education, medical care and social security system to promote increased participation in these programs.

## Supporting information

S1 File (DOCX)

S1 Questionnaire (DOCX)

S2 Questionnaire (DOCX)
