# Supplementary material for: Knowledge, attitude and willingness of different ethnicities to participate in cadaver donation programs
Source: PLoS One. 2020 Mar 12;15(3):e0229529. doi: 10.1371/journal.pone.0229529 (PMC7067454; doi:10.1371/journal.pone.0229529)
Supplement: S1 File — (DOCX) [file pone.0229529.s001.docx]

Dear Matthew Miller:

We wish to thank you for the time and effort you have spent reviewing our paper.

Motivated by your comments, we have deeply reconsidered the architecture of our

work and tried to fix all the problems you mentioned.

1. Please ensure that the author list and affiliations are correct on the title page of your manuscript, and that your author contributions, competing interests, and financial disclosure are correct as listed below. All of these sections will be indexed in PubMed and published by PLOS ONE as you have written them. Please email plosone@plos.org if any changes to this content need to be made.

**Response:** We ensure that the author list and affiliations are correct

2. Please confirm that all information in your Funding Information is also present in your Financial Disclosure. Only the Financial Disclosure section will be published alongside your article to describe your funding.

Funding Information:

National Outstanding Youth Science Fund Project of National Natural Science Foundation of China 81460492 Mr. zhongming Li

Basic Research Grant of Yunnan Province 2017FE468-189 Mr. zhongming Li

Teaching Reform and Teaching Research of Kunming Medical University 2019-JY-Y-00 Mr. zhongming Li

**Response:**

It is my mistake.

Funding Information:

~~(National Outstanding Youth Science Fund Project of National Natural Science Foundation of China),~~ expression is inaccurate and should be changed to (the National Natural Science Foundation of China)81460492 Mr. zhongming Li

Basic Research Grant of Yunnan Province 2017FE468-189 Mr. zhongming Li is ringt.

Teaching Reform and Teaching Research of Kunming Medical University 2019-JY-Y-00 Mr. zhongming Li is right.

4. We note that there is a discrepancy with your funding between the Financial Disclosure statement and Funding Information section in the submission form. Currently in the online submission form, your Financial Disclosure statement reads as follows:

The author(s) received no specific funding for this work.

However, I see that your Funding Information section contains the following additional funder(s):

National Outstanding Youth Science Fund Project of National Natural Science Foundation of China 81460492 Mr. zhongming Li

Basic Research Grant of Yunnan Province 2017FE468-189 Mr. zhongming Li

Teaching Reform and Teaching Research of Kunming Medical Universit 2019-JY-Y-005 Mr. zhongming Li

Please know that we will only publish the Financial Disclosure statement alongside your article. With your approval, we would like to update your Financial Disclosure statement in the online submission form to read as follows:

This work was supported by the National Outstanding Youth Science Fund Project of National Natural Science Foundation of China grant 81460492, Basic Research Grant of Yunnan Province grant 2017FE468-189, Teaching Reform and Teaching Research of Kunming Medical University grant 2019-JY-Y-005. The funders had no role in study design, data collection and analysis, decision to publish, or preparation of the manuscript.

Please confirm if this update is appropriate.

**Response:**

It is my mistake, expression is inaccurate.

This work was supported by ~~the National Outstanding Youth Science Fund Project of National Natural Science Foundation of China grant~~ (the National Natural Science Foundation of China)81460492, Basic Research Grant of Yunnan Province grant 2017FE468-189, Teaching Reform and Teaching Research of Kunming Medical University grant 2019-JY-Y-005. The funders had no role in study design, data collection and analysis, decision to publish, or preparation of the manuscript.

2. We note your current Data Availability statement: "All relevant data are within the manuscript and its Supporting Information files." However, it seems there are no data in your manuscript or your Supporting Information files. Please clarify whether there are ethical or legal restrictions against the public sharing of your minimal data set. PLOS ONE describes the minimal data set as that which is used to reach the conclusions drawn in the manuscript with related metadata and methods, and any additional data required to replicate the reported study findings in their entirety. This may include:

- The values behind the means, standard deviations and other measures reported

- The values used to build graphs

- The points extracted from images for analysis

**Response:**

In this study, all the subjects are provided with proper guidance of informed consent process. Verbal consent will be utilized in this study instead of obtaining the subject’s signature due to the concern of privacy issues from the standpoint of the participant. The verbal consent process has been approved by the Ethics Committee of Kunming Medical University.

For these ethical or legal restrictions reasons, we do not intend to publish details of the investigation to protect the personal privacy of the respondent (group). We are very disturbed and hope that the editors will understand and forgive.

6. Author Zhong ming Li is listed as zhongming Li in the online submission form; we are unsure which version is correct. If the name is incorrect in the manuscript, please correct this. If the name is incorrect in the submission form, please let us know so we can correct the author’s profile for you. Please note that the use of middle initials/names should be consistent among the manuscript’s author list, the author list in the submission form, and the Author Contributions initials.

**Response:**

It is my mistake.

zhongming Li is right, Zhong ming Li is worng.
