# Supplementary material for: Knowledge, attitude and willingness of different ethnicities to participate in cadaver donation programs
Source: PLoS One. 2020 Mar 12;15(3):e0229529. doi: 10.1371/journal.pone.0229529 (PMC7067454; doi:10.1371/journal.pone.0229529)
Supplement: S1 Questionnaire — (DOCX) [file pone.0229529.s002.docx]

| **Questionnaires**  **Hello:**  **This is a survey on citizens' Knowledge, attitude and willingness about body donation. This questionnaire is anonymous. There is no right or wrong answer. This survey is only for research purposes. All your information will be kept confidential and will not affect you in any way.**  **Please fill in or check the appropriate item for you, thank you!**  **Research Group**  **1.program (Table 1)**  1) Age:( )  2) Gender: 口 Female 口 Male  3) Ethnicity: ( )  4) Education: 口 Uneducated 口 Primary School-Senior High School 口 Junior college or above  5) Religion: 口 Buddhism 口 Taoism 口 Christian 口 Islam 口 Catholic 口 Other 口 None  6) Occupation: 口 Public officer 口 Technician 口 Agricultural personnel  7) Monthly income: 口<2000元 口2000～5000 口 >5000  **2. Knowledge of body donation program (Table 2)**  1) Do you know that citizens have the right to make body donations?  口 No, I have never heard of that  口 I have heard of that but not sure  口 Yes, I know  2) Do you know anyone who was a body donor? 口 Yes 口 No  3) Do you know the procedure of body donation? 口 No 口 Yes  4) Do you know any registries for body donation? 口 No 口 Yes  5) What do you want to know most about body donation?  口 Legal and Ethical Topics 口 Psychosocial adaptation and care for family of donor  口 Body donation procedures  **3. Attitudes toward body donation program (Table 3)**  1) Body donation is beneficial to the progress of medicine research and anatomical sciences.  口 Agree 口 Disagree 口 Neutral  2) Body donation program should be registered at the time of obtaining a driver’s license.  口 Agree 口 Disagree 口 Neutral  3) Body donation should be recognized by issuance of honorary certificates from the government.  口 Agree 口 Disagree 口 Neutral  4) Financial compensation violates the original intention of the donor.  口 Agree 口 Disagree 口 Neutral  5) Financial compensation will increase commercialization of body donations.  口 Agree 口 Disagree 口 Neutral  **4. Please check on the item that best describes your situation（Table 4）**  1) Would you be willing to grant permission for body donation?  口 Agree 口 Disagree 口 Don’t know  2) Whose suggestion will you take when you decide to grant permission by signing donation consent? 口 Myself 口 Family members 口 Friends 口 Colleagues 口 Other  3) Body donation is not against your religion/faith. 口 Agree 口 Disagree 口 Don’t know  4) As long as the donation consent was signed, the bereaved family has no right to prevent the body donation after the death.  口 Agree 口 Disagree 口 Don’t know |
| --- |
