# Supplementary material for: Knowledge, attitude and willingness of different ethnicities to participate in cadaver donation programs
Source: PLoS One. 2020 Mar 12;15(3):e0229529. doi: 10.1371/journal.pone.0229529 (PMC7067454; doi:10.1371/journal.pone.0229529)
Supplement: S2 Questionnaire — (DOCX) [file pone.0229529.s003.docx]

| **调 查 表**  **您 好：**  **这是一份关于公民对遗体捐赠相关知识的调查。本问卷采用不记名方式，答案没有对错之分，本次调查只为课题研究所用，我们承诺：您的一切信息都将保密，不会对您产生任何影响。**  **请按项目如实填写或在适合您的项目上打“√”，非常感谢！**  **遗体捐赠认知、态度、意愿课题组一、一般资料（表1）**  1、年龄：（ ）  2、性别：口女 口男  3、民族：（ ）  4、文化程度：口未受过教育 口小学～高中 口专科以上  5、有无宗教信仰：口佛教 口道教 口基督教 口伊斯兰教 口天主教 口其它 口无  6、职业：口公职人员 口技术人员 口农业人员  7、月收入：口<2000元 口2000～5000 口 >5000  **二、遗体捐赠认知状况（表2）**  1、是否知道公民有实施遗体捐赠的权利： 口从未听说 口曾经听说过，但不确定 口知道  2、周围是否有人实施过遗体捐赠行为：口有 口无  3、是否知道遗体捐赠程序： 口不知 口知道  4、是否知道遗体捐赠登记机构： 口不知 口知道  5、您最想加强哪些遗体捐赠知识：  口法律与伦理议题 口捐赠家属心理社会调适与照护 口捐赠流程相关知识  **三、遗体捐赠态度（表3）**  1、死后把遗体捐出可以造福人类： 口 同意 口不同意 口不确定  2、驾照领取与签署遗体捐赠志愿书同步： 口 同意 口不同意 口不确定  3、政府或相关机构应向捐赠者及家属颁发荣誉证书：口 同意 口不同意 口无所谓  4、向捐赠者及家属实施经济补助有违捐赠者初衷：口 同意 口不同意 口不确定  5、经济补偿致使遗体捐赠商品化可能性增加：口 同意 口不同意 口不确定  **四、请根据真实情况，在最符合您情况的一项上打“√”。（表4）**  1、您是否愿意捐赠遗体：口 同意 口不同意 口不确定  2、您签署遗体捐赠自愿书时，主要考虑谁意见？：口自己 口家人 口朋友 口同事 口其它人  3、遗体捐赠与我的宗教信仰并不违背：口 同意 口不同意 口不确定  4、只要生前签署遗体自愿捐赠愿意书，亡故后家属也无权阻止捐赠行为实施：  口 同意 口不同意 口不确定 |
| --- |
